# Acceptability of a real-time notification of stress and access to self-help therapies among law enforcement officers

**DOI:** 10.1186/s12889-021-12423-y

**Published:** 2022-01-06

**Authors:** Katelyn K. Jetelina, Rebecca Molsberry, Lauren Malthaner, Alaina Beauchamp, M. Brad Cannell, Trina Hall, Ed Fowler, Lonzo Anderson

**Affiliations:** 1 Department of Epidemiology, Human Genetics, and Environmental Sciences, University of Texas Health Science Center at Houston, School of Public Health, 6011 Harry Hines Blvd, Dallas, TX USA; 2 Dallas Police Department, Dallas, TX USA; 3 Rockwall Police Department, Rockwall, TX USA

**Keywords:** Police, Mental health, Stress, Technology, Smart watch

## Abstract

**Background:**

Law enforcement officers (LEOs) are exposed to chronic stress throughout the course of their shift, which increases the risk of adverse events. Although there have been studies targeting LEO safety through enhanced training or expanded equipment provisions, there has been little attempt to leverage personal technology in the field to provide real-time notification of LEO stress. This study tests the acceptability of implementing of a brief, smart watch intervention to alleviate stress among LEOs.

**Methods:**

We assigned smart watches to 22 patrol LEOs across two police departments: one suburban department and one large, urban department. At baseline, we measured participants’ resting heart rates (RHR), activated their watches, and educated them on brief wellness interventions in the field. LEOs were instructed to wear the watch during the entirety of their shift for 30 calendar days. When LEO’s heart rate or stress continuum reached the predetermined threshold for more than 10 min, the watch notified LEOs, in real time, of two stress reduction interventions: [1] a 1-min, guided breathing exercise; and [2] A Calm app, which provided a mix of guided meditations and mindfulness exercises for LEOs needing a longer decompression period. After the study period, participants were invited for semi-structured interviews to elucidate intervention components. Qualitative data were analyzed using an immersion-crystallization approach.

**Results:**

LEOs reported three particularly useful intervention components: 1) a vibration notification when hearts rates remained high, although receipt of a notification was highly variable; 2) visualization of their heart rate and stress continuum in real time; and, 3) breathing exercises. The most frequently reported type of call for service when the watch vibrated was when a weapon was involved or when a LEO was in pursuit of a murder suspect/hostage. LEOs also recollected that their watch vibrated while reading dispatch notes or while on their way to work.

**Conclusions:**

A smart watch can deliver access to brief wellness interventions in the field in a manner that is both feasible and acceptable to LEOs.

**Supplementary Information:**

The online version contains supplementary material available at 10.1186/s12889-021-12423-y.

## Background

Law enforcement officers (LEOs) are at an increased risk of physical, psycho-social, and anticipatory stress [1–3] compared to those with other occupations [4–7]. Stress can be cyclical throughout the course of a shift, a work week, or a career, which increases the risk of adverse events like LEO injury, excessive use-of-force, and death [8, 9]. LEO’s chronic exposure to stressors also influences physical and mental health conditions like anxiety, depression, post-traumatic stress disorder, burnout, and sleep disorders [10, 11], as well as absenteeism from duty [12]. On average, 30,990 nonfatal injuries resulting in days away from work are reported for LEOs annually [4], leading to significant productivity losses and increased costs for law enforcement agencies [13, 14].

There has been a breadth of recent research seeking to improve the occupational health and safety of LEOs through enhanced training or expanded equipment provisions [15–18]. For example, LEO counseling and training has been demonstrated to improve officer stress [19]. Tailoring shift work [20] and team-based health promotion [21] has significantly reduced stress and injury rates among LEOs too [22]. Furthermore, the introduction of mindfulness-based interventions has shown improvements to stress and quality of life among LEOs [23–25]. However, there has been little attempt to leverage technology in the field to provide real-time notification of LEO stress.

Prior research has found that stress can be quantitatively detected by physiological measures [26–28] among police officers while on-duty, and specifically, detected by personal technology [29]. This study, which leveraged FitBits, found high rates of smart watch device utilization, with 95% of the LEOs wearing the watches for more than 30 consecutive days [29]. Additionally, researchers mapping LEO stress via smart watches found that officers’ heart rates increased significantly during calls for service, particularly during higher priority calls [30]. Researchers have also found that smart watch technologies are an acceptable way of increasing police officer physical activity [31].

A natural next step in this line of research is to investigate whether a smart watch-based intervention can break a LEO’s cycle of consecutive stress. This study assesses the preliminary acceptability, through qualitative interviews, of a 30-day smart watch intervention to notify and alleviate consecutive stress among LEOs. We hypothesize that the intervention, fully or partially, will be acceptable to LEOs, which can then be used to design a large, effectiveness trial.

## Methods

### Setting

The State of Texas has 150 law enforcement agencies, 61 of which cover the 2600 mile^2^ Dallas-Fort Worth geographic area. For the current study, we sampled individual LEOs from two Dallas area law enforcement agencies: 1) large urban department with over 3000 LEOs; and, 2) suburban department with 85 LEOs.

### Study population

Because of financial and time restraints from the funding source, we limited our sample to 22 LEOs (11 urban and 11 suburban) for this project. Eligible participants were 1) aged 18 or older; 2) currently employed (i.e. LEOs holding a Texas Commission on Law Enforcement Officer Standards and Education license); and, 3) finished with their probationary period (at least one-year post academy).

For the urban police department, we targeted two different divisions known for high occupational stress. The first was a team that specialized in criminal and drug complaints, in which 5 LEOs responded to an invitation email with interest in the project. The other 6 urban LEOs were from a patrol shift (hours of 11p – 8a) in a high crime area. For this shift, an email was distributed to 66 patrol LEOs, with exactly 6 interested LEOs who responded (9%).

**Fig. 1:**
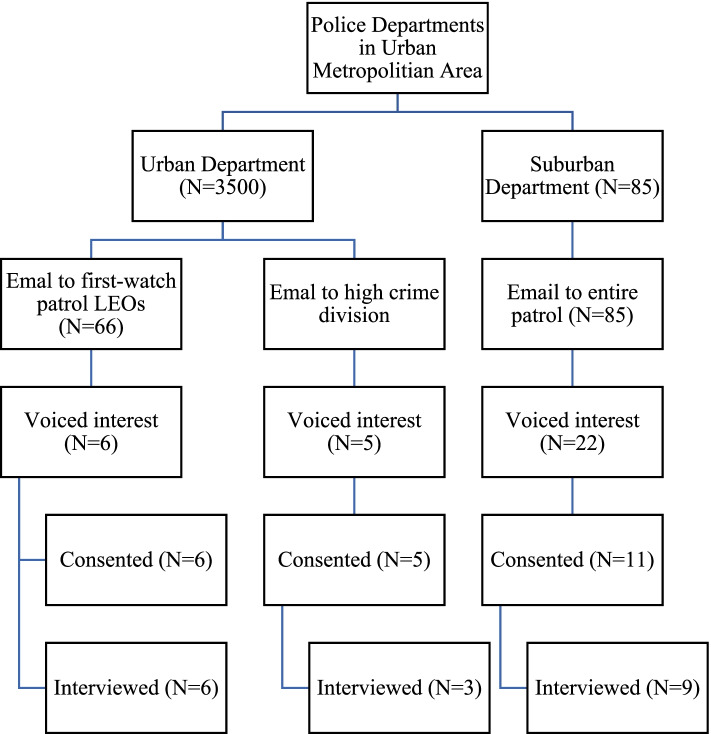
PRISM Diagram

For the suburban department, an invitation was emailed to 85 LEOs, of which 22 LEOs voiced interest in participating (26%). From this list, we used stratified random sampling based on tenure and position to select the final sample size (*n* = 11). See Fig. 1 for the PRISM diagram.

### Implementation

**Fig. 2:**
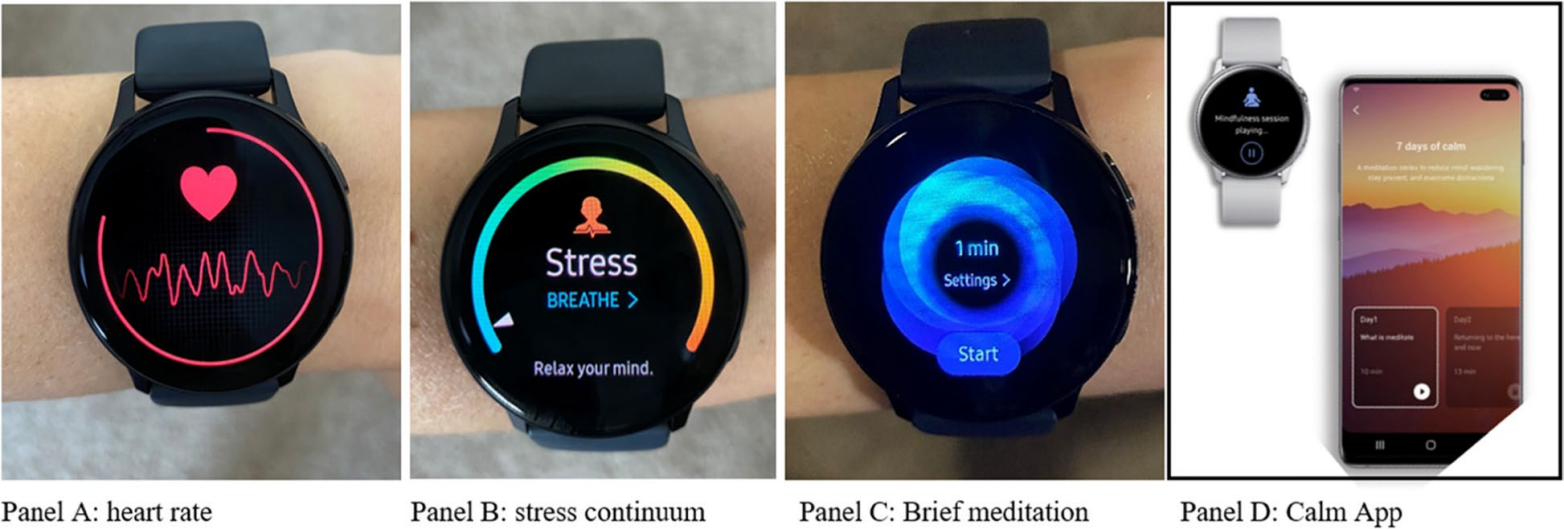
Smart watch capabilities

Once LEOs were recruited, there was a four-step implementation process. First, an orientation presentation was emailed to LEOs, which detailed emotional wellness tips from a licensed psychologist for managing stress throughout a shift. Along with best practice recommendations, the presentation outlined the role of the intervention in targeting LEO stress and introduced to the heartrate monitor and stress continuum monitor build into the watch (Fig. 2). The heart rate displayed real-time heart rate of the wearer. The stress continuum was another visual display of heart rate, which was standardized and ranged from 1 to 100. During the presentation, LEOs were also introduced two wellness options to use in the field: 1) A 1-min meditation breathing exercise that was already built into the smart watch, and 2) the Calm app™, which provided a mix of guided meditations and mindfulness exercises for LEOs needing a longer decompression period. The Calm app was downloaded on their phone and smart watch during the in-person set up session (see more below). Watch capabilities are presented in Fig. 2. At the end of the presentation, a link was provided to LEOs to sign up for a watch set-up time.

Second, participants attended an in-person set-up session for consent and to watch configuration. At this session, we activated participants’ watches, measured their resting heart rates (RHR), and educated them on the brief wellness interventions available on the watch for use in the field. LEOs were randomly assigned to a predetermined threshold (50, 60, 70%, or 80% higher than RHR) for intervention notification. The purpose of randomizing thresholds was for two reasons. First, there is limited research available on a universal elevated heart rate indicative of high stress due to several factors that impact heart rate. Previous research has found that a single measurement of heart rate is only useful when it’s well out of the expected range of normal [32]. Second, as this was a pilot study, we wanted to assess various thresholds to identify the percentage above RHR most beneficial for LEO notification (regular but not over-notification of high stress). Participants also completed a brief, 8-item demographic survey.

Third, LEOs were instructed to wear the watch during the entirety of their shift for 30 calendar days. When participants’ heart rates or stress continuums reached their predetermined threshold for a period of ten minutes or greater, the watch notified the LEOs that they were experiencing heightened physical stress via a vibration mechanism on the watch. After notification, two mental health interventions were available in real time through the watch: (1) a 1-min, meditation breathing exercise; and (2) the Calm app™. Watch capabilities are displayed in Fig. 2.

Finally, 30-day post-baseline, interviews were scheduled between August–September 2020 and conducted virtually on WebEx due to COVID19 restrictions. The semi-structured interviews lasted approximately one hour and began with general discussion of the project. During the interview, LEOs were asked about intervention components (Appendix A). Data collection for this study was approved by the Center for the Protection of Human Subjects at the University of Texas Health Science Center (HSC-SPH-20-0126). Informed consent to participate in the study was obtained from all participants.

### Analysis

All interviews were audio recorded and professionally transcribed. A multi-disciplinary team used a three-step approach to analyze qualitative data [33]. First, the research team collectively read transcripts collected from each interview to develop a deeper understanding of the discussion. Through this process, a deductive codebook was created to label text. We used these codes in group analysis sessions until we reached stability. Second, text was individually coded by the members of the research team. We grouped emerging findings into categories of themes using an immersion-crystallization approach [34], which included inductive thematic identification. Third, transcripts were read by a second and third coder and coding inconsistencies were discussed and resolved by consensus. Nvivo 12.0 software (QSR International Pty Ltd.) was used for all coding, organization, and data reduction.

## Results

Table 1 displays the demographics of study participants. Briefly, 86% were male, 73% were non-Hispanic White, and 55% were a college graduate. More than half of LEOs were married (60%) and 32% had a history of military service. Average tenure was 9 years (Standard deviation [SD] = 5), ranging from 3 to 18 years. The average age of study participants was approximately 36 years (SD ± 10.6). There were no statistical differences in those that participated in the intervention (*n* = 22) compared to those that participated in the debrief interviews (*n* = 18).

**Table 1:** Sample description

	***Intervention*** *n* = 22 N (%)	***Interviews*** *n* = 18 N (%)
Age (years); mean, (SD)	36.4 (10.6)	34.4 (9.3)
Sex		
Male	19 (86)	15 (83)
Female	3 (14)	3 (17)
Race/Ethnicity		
White NH	16 (73)	15 (83)
Black NH	1 (5)	0 (0)
Hispanic	5 (23)	3 (17)
Education		
1–3 Years of College	9 (41)	7 (39)
College graduate	12 (55)	10 (56)
Master’s graduate or higher	1 (5)	1 (6)
Current marital status		
Married	13 (60)	10 (56)
Divorced	4 (18)	4 (22)
Never married	5 (23)	4 (22)
Tenure (years); mean, (SD)	9.1 (5.0)	8.56 (4.8)
Consistently rode with a partner	4 (18)	4 (22)
Military experience (Yes)	7 (32)	5 (28)
Police department		
Urban	11 (50)	9 (50)
Suburban	11 (50)	9 (50)
Resting heart rate (beats/min); mean, (SD)	75.4 (9.4)	73.7 (7.2)

*SD* standard deviation; *NH* non-Hispanic; *min* minute

Three components of the intervention positively influenced LEO stress: 1) receiving a vibration notification; 2) visualizing their heart rate and stress level on the watch; and, 3) participating in short breathing techniques in the field. Evidence for each theme is discussed below.

### Vibration

LEOs were notified, by a vibration on their watch in real-time, when their heart rate reached a pre-determined threshold (ranging from 50 to 80% above RHR) for ten consecutive minutes. After the 30-day trial, 50% of the LEOs self-reported that their watch vibrated during their shift (Table 2). All participants at the 70% threshold level self-reported that their watch vibrated during the trial period.

**Table 2:** Vibration notification patterns across set heart rate threshold

*Threshold above resting heart rate*	*LEOs assigned to threshold level* *N (intervention); N (interviewed)*	*# LEOs reported vibration (%)*
50%	6: 4	2 (50%)
60%	6: 6	2 (33%)
70%	5: 4	4 (100%)
80%	5: 4	1 (25%)

During the debriefing interviews, LEOs were asked to describe the times they remember their watch vibrated. Table 3 displays the frequency in which LEOs reported situations (types of calls or other times) in which the watch vibrated. The most frequently reported type of call for service was when a weapon was involved or when a participant was in pursuit of a murder suspect/hostage. LEOs also remembered that their watch vibrated while reading dispatch notes (in anticipation of a call) or when they were on their way to work (before shift).

**Table 3:** Situations in which participants reported a vibration (*n* = 8)

	N of LEOs (%)
***Type of call***	
Weapon involved	6 (75)
In pursuit of murder suspect/hostage	5 (63)
Assisting another LEO in trouble	3 (38)
Almost hit by a car	1 (13)
Child abuse	1 (13)
***Other times***	
Reading dispatch notes on the way to a call	7 (88)
Driving to work (before shift)	3 (38)
Crowd	2 (25)
Training a rookie	1 (13)
Supervisors	1 (13)
Working out	1 (13)

*LEO* Law Enforcement Officer

Among LEOs that indicated experiencing a vibration (*n* = 8), 100% of LEOs reported that the notification system had a positive impact on stress awareness. One LEO stated:

“*Just feeling that buzz and knowing that I need to maybe take some deep breaths, knowing that [my heart rate] is high and trying to get it back down…that would definitely help if officers had that opportunity to be buzzed while it was during their shift*”.

Two other LEOs stated:

“*It was helpful, but you kind of feel your heart rate or your pulse going up [without the watch] but I guess the watch would remind you. The watch may catch your pulse going up maybe before you do and maybe you can start deescalate when you’re notified*”;

And,

“*Having something that tells you, hey, this is happening, and then gives you some way to mitigate it from there is helpful*”.

Among officers that remembered a vibration (*n* = 8), 100% of LEOs reported that the vibration notification system did not distract from duties in the field. “*It’s different ‘cause I never had a smartwatch before, so it’s different to see your notifications of messages coming in. But no more different than my phone going off in my pocket. I’ll look at it later*”. Another LEO noted the vibration did not “*take my attention off of what I was doing. I mean I’m sure there’s many of times where it vibrated and I kind of just ignored it really*”. Another LEO noted that the vibration wasn’t a distraction and, in fact, “*it helped, if anything*”.

Among officers that didn’t remember the vibration (*n* = 8), two LEOs noted that the buzzing might be distracting for officers in the field. But it really depends on frequency. As one LEO said, “i*f the watch is constantly buzzing, it’s kind of hard to get away from everything”.*

### Visualization

LEOs reported favorably to the stress continuum function on the watch (Fig. 2, Panel B), and specifically the ability to visualize their stress: “*I have often checked it, especially after a high stress type thing, or a fight, or intense situation at work, and I’d look at it and see I’m elevated, and try to calm myself down too*.” “*I tried to be more in the moment and you know thinking about if my stress level is high. So, I think [visualizing the stress continuum] did help*”.

This stress continuum function helped initiate interventions in the field. As one LEO reported: “*When I realized that my stress was in the red, that’s when I started doing the breathing techniques to bring it back down*.” Another LEO stated “*I saw the stress level spike up, so I was just like, ‘Let me try [breathing]’ and it actually worked*”.

LEOs also preferred visualizing the heart rate monitor (Fig. 2, Panel A). “*Watching my heart rate fluctuate, decrease, so I was able to manage it. It was interesting to be able to see that*”. Another LEO “*checked it often, especially after a high stress type thing, or a fight, or intense situation at work, and I’d look at it and see I’m elevated, and try to calm myself down*”. And, “[*Heart rate monitor] definitely helps you kind of like think about it and be like, hey, you need to relax or do something to get it down to normal*”.

LEOs tended to use the heart rate monitor more than the stress continuum, though. As one LEO stated:

“*I look at the heart rate more often 'cause the watch face I used had the heart rate built into the watch face. So, I can quickly glance and see it. With the stress, I'd have to navigate to that particular page. But every once in a while, I would look at it, and then after those high stress calls I would go into the app, get it there, and see where it spiked too, and saw where it was the highest*.”

### Breathing techniques

Breathing exercises worked in reducing real-time, acute stress. LEOs liked how “*it actually gives you the time that you need to take to breath*” and found “*it was one of the main things that helped me*”. After calls, some LEOs stated that they used the breathing exercises as a “*game*” between calls for service. One said: “*It was interesting to sit and do some different types of breathing to see if I can make the [stress app] come back down and then how low I could make it go just sitting there*”. Another LEO reported that they “*would try the breathing. And I would also just walk away from the scene. I could feel myself kind of relaxing a little bit, stepping away from the scene*”. Another LEO stated:

“*My heart rate would start to elevate, whether it was because I was getting irritated, or in anticipation of whatever it was, or whatever the feeling was, and then it was kind of interesting to sit and do some different types of breathing to see if I can make it come back down and then how low I could make it go just sitting there.”*

Interestingly, though, some LEOs did not prefer using the applications provided by this research project. “*I did some breathing. I didn’t necessarily try the one on the watch*”. Another LEO reported that they “*had done a class and found that was one of the main things that helped [him]*”. One LEO agreed by stating: “*I did the structured breathing through the watch a couple of times and then more often than that, I just did it on my own where I would focus on just breathing in four or five seconds*”. Once we prompted the LEOs where they learned these breathing exercises, a few of the LEOs reported taking a prior meditation class through the department and/or prior military training in combat breathing.

## Discussion

Prior research has both described high unmet mental illness needs [8, 35–38] and found that personal technology, like FitBits, can detect psychological stress among LEOs [29]. This study subsequently found that personal technology can also, then, bring awareness to stress through brief, real-time stress reduction practices in the field in a manner that is acceptable to LEOs. This is particularly important because LEOs have continuous exposure to stress throughout their shift and have voiced difficulty getting back to “zero” before their next call for service [9]. This continuous stress impacts job performance and adverse events (e.g. injury; use of force; officer involved shootings [11];). LEOs particularly endorsed the visualization of a heart rate monitor and vibration notification of sustained high-stress. Importantly, the vibration was not a distraction while in the moment of a high-stress call, but rather reminded LEOs that they were stressed and should intervene after the situation was under control.

Only 50% of LEOs remembered the watch vibrating, of which 100% of LEOs assigned to a 70% above RHR remembered vibrations. This suggests that 70% above RHR may be the optimal threshold for future interventions, which is consistent with prior research [26–28]. However, there were no meaningful patterns above and below the 70% threshold. For example, we would hypothesize that those with a lower threshold would remember vibration more than those with a high threshold. While this could be attributed to recall bias, this also could have been due to the way we measured RHR. LEOs were instructed to pick up their watch at the station, where stress is already high by just being in the building, as mentioned by study participants. Also, for participant convenience, times were scheduled before or after a shift. The combination of the aforementioned processes could have provided biased RHRs. Future research should measure RHR at home, prior to shift at the department, and after shift for a more accurate baseline.

While LEOs found breathing after high-stress calls helpful, interestingly, LEOs did not prefer the breathing exercises provided to them. LEOs preferred even briefer exercises for in the field. For example, preferred 5 s breathing exercises rather than 1-min or 10-min exercises. Also, we discovered that many LEOs in our sample had prior mindfulness training and combat breathing training.

Future research is needed to assess the effectiveness of this brief, field intervention in improving clinical mental health indicators over time, like depression, anxiety, PTSD, and suicide ideation. There were many lessons learned from this pilot that can be applied for future, larger trials. First, to accommodate LEOs, we collected baseline heart rate at the station before their shift. But, in this study we found the strong impact of anticipatory stress on LEOs on their way to the station. This likely means their baseline heart rate at work is not their “true” baseline. In the future, teams should collect baseline heart rate of LEOs several times at home. Second, it’s important to advocate for participant-level data with personal technology companies. This would both help measure compliance and psychological shifts throughout the work day. Third, it’s apparent that the age of an officer enhances or impedes set-up for smart watches and smart phones. For scalability, onboarding a large proportion of LEOs varying in age may be challenging and innovative solutions should be explored.

### Limitations

Results should be considered in light of several limitations. First, this qualitative study was small. However, we sampled over two police departments, which helps increase generalizability of findings. Second, sampling bias may be present due to convenience sampling among the urban LEOs. For example, LEOs that were interested in an intervention like this, may be systematically different than LEOs who would not participate in this intervention. Sociodemographically, though, participants did reflect that of the departmental general population. Third, we did not collect information on the mental and physical health of LEOs, which may differentially influence acceptability of intervention implementation. Finally, and unfortunately, we didn’t have access to the watch quantitative data on the back end, so we were dependent on self-reported compliance. One hundred percent of participants reported wearing their watch for the duration of the study. While there is the possibility of desirability bias with qualitative data, our previous research with the same target population found a 95% compliance with smart watches using quantitative data [29]. Future research should further explore how to reduce the barriers to accessing technological based metrics from technology companies.

## Conclusion

A smart watch can deliver access to brief wellness interventions in the field in a manner that is both feasible and acceptable to LEOs. Future research is needed to assess the effectiveness of this field intervention. In addition, it would be interesting to compare whether this intervention replaced or reduced prior, negative stress coping skills (e.g.*,* alcohol consumption), which would require a larger, longitudinal study.

## Supplementary Information

**Additional file 1.**

## Data Availability

The datasets used and/or analyzed during the current study are available from the corresponding author on reasonable request.

## Abbreviations

RHR: Resting heart rate
LEO: Law enforcement LEO
SD: Standard deviation
NH: Non-Hispanic
