# Supplementary material for: Acceptability of a real-time notification of stress and access to self-help therapies among law enforcement officers
Source: BMC Public Health. 2022 Jan 6;22:32. doi: 10.1186/s12889-021-12423-y (PMC8740346; doi:10.1186/s12889-021-12423-y)
Supplement: Supplementary file 1 — Additional file 1. [file 12889_2021_12423_MOESM1_ESM.docx]

# **Appendix A: Interview Guide**

Moderator: I want to know what you think. There are no right or wrong answers to the following questions, and all of your opinions are valuable.

First off, I would like to hear about the Smart Watch. Did you use it at all? Did it work well?

- Were the smart watch and associated apps easy to use?
- Did the watch distract or interfere with any calls?
- Has your understanding of your own stress levels changed over the past month?

What were your opinions on the heart rate alert and stress widgets?

- Were you aware of high-stress prior to receiving the notification?
- What did you do immediately following the intervention notification?
  - What coping techniques did you engage in following a notification of a high-stress response?
- Do you believe the time from increased stress response to notification was appropriate?
- Was either the heart rate tracking or stress tracking more useful to you?
- Were any of the other functions of the watch helpful for managing your health?

We are thinking about hooking up more officers with these smart watches. Do you think that’s a good idea?

- Do you think this intervention would be better suited for some officers vs. others?
- How could this intervention be expanded to better deal with the occupational stress of officers?
- How could leadership encourage buy-in from officers for this intervention?
